# Review of 'Ethics and AIDS in Africa: The Challenge to Our Thinking' by Anton A. van Niekerk and Loretta M. Kopelman (Eds)

**DOI:** 10.1186/1747-5341-2-1

**Published:** 2007-03-27

**Authors:** Stephanie A Nixon, Nkosinathi Ngcobo

**Affiliations:** 1 University of KwaZulu-Natal, Health Economics and HIV/AIDS Research Division, University of KwaZulu-Natal, Westville Campus, J Block, Level 4, University Road, Westville, Durban, South Africa; 2 University of Toronto, Department of Physical Therapy, 160-500 University Avenue, Toronto, Ontario, Canada

## Review

In 2004, bioethicist Michael J. Selgelid decried:

Given the magnitude of the AIDS catastrophe in Africa – and given that the causes and consequences of this crisis directly involve issues of justice – it is surprising that, to date, no major books addressing AIDS in Africa have been produced by (those who primarily consider themselves to be) bioethicists. As this is one of the most important issues of our time, the lack of bioethics literature is unfortunate and reflects poorly on the discipline of bioethics [1].

Anton A. van Niekerk of the University of Stellenbosch in South Africa and Loretta M. Kopelman of East Carolina University in the United States tackle Selgelid's challenge in their co-edited anthology of articles called, *Ethics and AIDS in Africa: The Challenge to Our Thinking *[2].

The book is not only about Africa but, importantly, draws heavily on the work of African scholars, which is significant in a field like bioethics that tends to be dominated by academics in North America and Europe. The contributions here and elsewhere from such African scholars as Godfrey Tangwa, Keymanthri Moodley and Solomon Benatar are required reading for Western ethicists concerned with international issues and Africa in particular.

Bioethics as a discipline tends to bring together scholars from a range of fields; so too does this anthology gather ideas from multiple perspectives including philosophers, economists and public health specialists. The book grew out of a special edition of the *Journal of Medicine and Philosophy *that was guest edited by van Niekerk and Kopelman in 2002. Approximately half of the articles in the current anthology were originally published in that special edition, which is a testament to the enduring value of their ideas four years later but also puts the content at risk of becoming outdated which is the case in places.

The chapters cover issues ranging from research ethics to public health ethics to metaethics, and consider the experiences of not only adults, but also children and infants. Several themes run throughout the book, including the contrast of 'Western liberalism' with 'Africa communitarianism', and the related ethical challenge of balancing risks to individuals with benefits to society as a whole. However, no book can do everything; a particular shortcoming in this book is its insufficient treatment of gender which is imperative for analysis of the HIV/AIDS pandemic in Africa.

Most importantly, van Niekerk and Kopelman's anthology provides us with a starting point on which to build a more robust body of scholarship on the ethical dimensions of AIDS in Africa. As noted by Van Niekerk, "HIV/AIDS is the most serious health and social crisis and challenge that has ever befallen Africa" (p. 141). Tangwa further notes,

"While the prospect of the possible annihilation of the entire human race by the HIV/AIDS pandemic is indeed far-fetched, that of erasing the African continent of its human inhabitants is not too far from the realm of possibility" (p. 185-6)."

This book is a timely, if not overdue, contribution to the field.

This review is organized into three sections. First, we provide an overview and critical analysis of the 13 chapters in *Ethics and AIDS in Africa*, highlighting strengths as well as area for further debate. We then narrow in on particular themes that have emerged throughout the anthology or are conspicuous by their absence, such as gender and human rights. The review finishes with a call for the development of a coherent ethical framework for comprehensively conceptualizating the ethical dimensions of AIDS in Africa and puts forth one possible structure for meeting this challenge.

## Part 1. Overview of chapters

### Characterizing the problem: overview of chapters 1 to 3

Despite the promise of ethics in the title of this book, the reader has to wait until the fifth chapter to encounter material that explicitly engages ethics theory. In the early chapters, the authors characterize HIV/AIDS in Africa and offer economic and pragmatic arguments for what ought to be done. The link to ethics is an implicit consequentialist assumption about the ethical imperative to act effectively, based on which the various authors propose economic, pragmatic and other arguments to guide action. In chapters 1 and 2, Whiteside and his colleagues paint an appropriately grim picture of the pandemic in Africa and discuss challenges around the use and misuse of AIDS data.

Chapter 3 is a beautifully argued synopsis of the main points from Nicoli Nattrass's book, *The Moral Economy of AIDS in South Africa*. She presents a compelling economic argument in favour of the immediate rollout of antiretroviral therapy (ART) in South Africa. She also discusses the catch-22 faced by people living with HIV who rely on state-sponsored disability grants as their main sources of income and, thus, dread the loss of those grants by becoming "too well" on ART. She argues that this horrendous choice between wellness and economic support for one's family can and should be overcome with a shift to a poverty-oriented basic income grant in South Africa, which she demonstrates is a feasible economic alternative.

### Social and political contexts: overview of chapters 4 and 5

In chapter 4, Anton A. van Niekerk, one of the two co-editors of the book, continues to set the stage through his description of selected social complexities that frustrate the response to HIV/AIDS in Africa. Among the most interesting contributions of this discussion is a reflection on how HIV/AIDS can reinforce and exacerbate existing prejudices about Africa. He reflects on Simon Watney's claim that:

"... Africa has been effectively demonized in a post-colonial discourse of perpetual catastrophe and unnatural disasters... 'Africans AIDS' thus condenses ancient fears concerning contagious disease, together with vengeful fantasies concerning 'excessive' sexuality, understood in essentially pre-modern terms as both the source and the cause of AIDS... (p. 59)."

Here, van Niekerk agrees with Loretta M. Kopelman's argument in chapter 13 regarding the counterproductive impact of the emphasis on blame and culpability for this disease, advocating instead that such misconceptions be abandoned and that energies be refocused on responding to HIV/AIDS in practical and compassionate ways.

This is also where we are introduced to the theme of the massive imbalance between wealth in Africa and the West. Van Niekerk relies heavily on the writing of Solomon Benatar (both here and in his other chapters) to point to the extraordinary inequity that characterizes this relationship. Benatar then tackles the issue head-on in chapter 5 where he identifies HIV/AIDS as a symptom of globalization, pointing to the kinds of corrections that are required to address not only HIV/AIDS but a range of other potentially disastrous by-products of the globalizing process. He notes:

"Structural adjustment programmes, debt repayments, cuts in foreign aid budgets (especially by the US), discrimination against African trade, increasing malnutrition and the cold-war activities of the great powers have all played a significant part in fanning the AIDS pandemic (p. 76)."

Importantly, for the first time in this book, Benatar also provides an ethics framework for considering these issues. He suggests that bioethics be expanded to consider three levels of concern as follows:

• micro-level concerns – ethical issues within doctor/patient and researcher/participant relationships

• meso-level concerns – ethical dimensions of order and justice within communities, including public health and the social good

• macro-level concerns – ethical issues at the level of the global economy and in relationships between nations that impact on human well-being

To date, the field of bioethics has focused largely on micro-level concerns to the exclusion of meso- and macro-level dimensions. To its credit, this book begins to redress this shortcoming with almost half of the chapters exploring what some are calling *public health ethics *[3], or the ethical dimensions of concerns with the health of collectives at the meso and/or macro levels. We shall return to Benatar's three-part framework at the conclusion of our review to analyze remaining gaps with respect to ethics and AIDS in Africa.

### Engaging with theories of justice: overview of chapter 6

After being lulled into expecting relatively accessible reading on the social and economic dimensions of HIV/AIDS, chapter 6 can arrive as a shock. Here we shift to a sophisticated engagement with ethics theory which will delight philosophers and terrify the rest of us that do not have the benefit of ethics training. This chapter was originally published in the South African Journal of Philosophy, which hints at its orientation. However, for those seeking a concise overview of Rawls' theory of justice as it may be applied to the global HIV/AIDS pandemic, this chapter is rewarding. Van Niekerk presents Rawls' theory of justice, followed by Buchanan's critique of Rawls, followed by his own critique of both insofar as their principles apply (or not) to HIV/AIDS in Africa. The result is a rich example of the kinds of analyses that would strengthen future volumes of this book.

We shall refer readers to the chapter for a full tour of the ideas; however, there is one controversial point worth raising here. At risk of oversimplifying, Buchanan argues that Rawls' ideas about justice (as explicated in his book, *The Law of Peoples*) ignores the "global basic structure', which Buchanan describes as "a set of economic and political institutions that has profound and enduring affects on the distribution of burdens and benefits among peoples and individuals around the world" (p. 96). Buchanan argues that this global basic structure can undermine the equality of peoples unless regulated by global distributive justice, which is in contrast to Rawls' approach based on charity. Van Niekerk then takes up this concern, stating: "I see no self-evident reason why the acceptance and execution of a 'duty of charity' has less of a moral force than the acceptance and execution of a 'duty of justice"' (p. 98).

It is our position, however, that the difference between obligations based on justice and those out of charity are profound and have dramatic consequences for responses to HIV/AIDS, particularly when considering the obligations of rich countries to support the global effort. The logic underpinning obligations is crucial because it can bear directly on the action that is subsequently required. For example, if a rich country is motivated by charity, then contributions of varying amounts of official development assistance, typically at well less than 0.7% GDP, can represent a satisfactory response. However, duties of justice can lead to different requirements. For instance, political philosopher Thomas Pogge describes a form of compensatory justice that understands moral obligations of rich nations as deriving from their complicity in the creation and maintenance of global poverty which fuels epidemics like HIV/AIDS [4]. As such, resultant actions from this duty can involve, for instance, redressing economic and trade policies that limit the capacity of poor countries to develop. A second critique of the limitations of charity-based duties is presented by Sckuklenk and Ashcroft regarding access to essential medicines in chapter 8.

### Debates on access to medicine: overview of chapters 7 and 8

The journey through this anthology gets even more interesting upon arrival at chapter 7 in which David Resnick (not to be confused with famed HIV denialist David Rasnick) tackles the major moral dilemma of insufficient access to medicines in poor countries. We describe this chapter as interesting because although Resnick purports to be analyzing the moral obligations of multinational pharmaceutical companies, by the end of the chapter his argument has transitioned into a warning for poor countries to become better customers by honouring pharmaceutical patents or else they may suffer in the long-term. Resnick begins with a balanced discussion of the role of big pharma in drug access issues, but then aligns his argument with the interests of industry by defending current drug pricing and patents. For example, he argues that: "... attempts to control or regulate profit margins could have drastic economic consequences for businesses that would restrict their ability to contribute to society" (p. 118). This is a hard claim to swallow for one of the most profitable industries in history whose 'contributions to society' seem unrelated in scope to the pattern of their economic gains.

Resnick advocates for poor countries to respect pharmaceutical patents. He acknowledges that countries with public health crises are legally entitled to use the flexibilities within the TRIPS agreement, but warns that this practice should be regarded "as a last resort" as opposed to a viable and legal opportunity to save millions of lives. He discusses ways "... to prevent nations from abusing their authority to override IPRs under the TRIPS agreement". However, it is now five years since the historic signing of the Doha Declaration (which clarified the legitimacy of public health and human rights trumping intellectual property rights in cases of emergency) and there remain worryingly few examples of countries taking advantage of the provisions. Why might this be? A common answer involves the asymmetrical power relations that exist between poor countries on one hand, and rich countries and their pharmaceutical lobbies on the other. These dimensions of the problem are not reflected in this chapter.

One final example is illustrative. Resnick argues that:

"It is hard to say exactly how much money pharmaceutical companies lose as a result of the failure to recognize patents globally. Industry representatives claim that they lose as much as ten percent of their profits this way, and it is likely that drug prices would be lower if the companies could take advantage of a larger market."

The implication is that drug prices are high because of markets lost to countries that do not respect patents. However, the reverse is more à propos: countries take advantage of the flexibilities afforded to them in TRIPs to issue compulsory licenses or parallel import drugs because the prices of drugs are prohibitively high.

In chapter 8, Udo Schuklenk and Richard Ashcroft approach the issue of patents and drug access from an alternative perspective. Whereas Resnick advised poor countries against "overriding patents" (p. 124), Schuklenk and Ashcroft frame the problem of access as worse in countries that have "restrictive patent laws" (p. 127). In further contrast to Resnick, they see compulsory licensing as the key strategy for beginning to reverse the problem of drug access for Sub-Saharan Africa. They present and reject three solutions that have been proposed for improving drug access – (a) donation of drugs from pharmaceutical companies, (b) price reductions, (c) public-private partnerships – before presenting their ethical rationale in favour of compulsory licensing as the way forward.

They reject both drug donations and price reductions on the grounds that these arrangements are fraught with conditions and time limits, and rely on the generosity of pharmaceutical companies for health care planning. Harkening to our earlier argument, they note a "moral distinction between charitable giving as voluntary and acting on duty" (p. 132). A further shortcoming is that the provider in these arrangements (i.e. the pharmaceutical company) can stop if the recipient (i.e. the country coping with an HIV crisis) is perceived to be ungrateful or undeserving, which in turn promotes an attitude of humility toward the giver and limits the ability of the recipient to set the terms of receipt. As such, Schuklenk and Ashcroft argue that these approaches are morally problematic for aretaic reasons related to the motives of the donor and character of the recipient, and on consequentialist grounds since they are not sustainable.

They then reject the bulk purchase of medicines through public-private partnerships because of their lack of success to date. They note that such efforts initiated by WHO, UNAIDS and UNICEF have demonstrated a confused internal logic because, in choosing to work with market mechanisms (e.g., bulk procurement), the agencies have assumed an approach that does not challenge industry's patent regime. However, the role of patents is to create a monopoly that prevents market mechanisms from working. Indeed, each of these three solutions accepts that industry will control the prices of medicines, meaning that all strategies rely on appeals to the moral obligations of pharmaceutical companies, which, Schuklenk and Ashcroft argue, has lead nowhere to date. All rely on charity, which usually fails us and is not a long-term strategy.

Compulsory licenses, they argue, serve to overcome these shortcomings. Intellectual property rights are designed to promote innovation in the public interest, but where they contravene public interest, this justification disappears. Schuklenk and Ashcroft propose that IPRs have been used as a diversion from the real issue of unaffordability of medicines. They also suggest that too much credence has been given to concerns about how social acts from moral obligations can compromise shareholders value and raise R&D costs without any evidence on the point. They thus find compulsory licensing to be one of the most important initiatives for addressing the gross lack of drug access in poor countries.

A key strength of anthologies is their capacity to include contrasting perspectives on a single issue. The dual consideration given to access to medicines in this anthology made for a more robust and spirited treatment of the subject. We would have welcomed the editors to have used this approach more broadly.

### Ethical dimensions of research in Africa: overview of chapters 9 to 12

In chapter 9, Anton A. van Niekerk provides a welcome review of the research ethics firestorm that began as a result of drug trials for prevention of mother-to-child-transmission (PMTCT) in Africa. In short, in 1994, researchers in the West demonstrated that a course of the drug, AZT, could dramatically reduce transmission of HIV from mother to child. However, the intervention was seen as prohibitively expensive for poor countries and clinically onerous, requiring a long time to be administered. Shortly thereafter, condensed regimens of AZT were tested in poor countries in Asia and Africa and were revealed to also be successful for PMTCT. Then, an even simpler and less expensive course of a different drug, Nevirapine, was demonstrated to be effective. The firestorm was ignited by an article published by Lurie and Wolfe [5] that denounced the trials in poor countries as unethical since they tested the drugs against placebos when the long course of AZT was already known to be effective. The central debate that emerged and which continues today involves the issue of the standard of care against which new interventions should be tested.

Van Niekerk accounts for the divergent views within this debate by drawing on the fundamental tension in the ethical approaches of deontology and consequentialism. Whereas deontologists are concerned with the same rules applying for all and the avoidance of double standards for rich and poor, consequentialists are concerned with the path that will provide the greatest justice to all. Van Niekerk ties this confusion to the appallingly slow rollout of PMTCT within South Africa drawing on critiques of the South African political leadership on the issue. We would also have welcomed a more complete overview of the debate over standard of care because of its impact on the field of research ethics as a whole.

Keymanthri Moodley describes HIV vaccine trials as "the next major ethical challenge in South Africa research circles" (p. 160) after PMTCT. As such, HIV vaccine trials are the focus of Moodley's chapter and the two that follow. In chapter 10, Moodley uses the four principles from the 1979 Belmont Report as a framework for examining HIV vaccine trial participation in South Africa. Although these principles – autonomy, beneficence, non-maleficence and justice – have received almost legendary status as the dominant ethical framework in bioethics, they were developed in the West and deserve to be scrutinized for their utility in African settings. Moodley is also concerned with the tension that exists between Western orientations of liberalism and individualism in contrast to African notions of communitarianism and *ubuntu*.

Within this context, he provides a useful critique of the Western approach to informed consent processes for participation in HIV vaccine research. Whereas the Western conceptualization views people as rational, individual and separate from others, African notions of personhood are relational, emphasizing reciprocity and interdependence. Furthermore, Moodley explains, the combination of illiteracy, language barriers and contrasting belief systems present a challenge to the competence requirement for informed consent. As he notes: "Too little protection of these subjects risks their exploitation; too much protection risks unjustified paternalism" (p. 167). He argues that in the aftermath of Apartheid in South Africa, many people who are competent will still relinquish their decision-making rights to authority figures. He explains that power asymmetries are particularly problematic when researchers and participants are from different racial groups.

Returning to the issue of HIV vaccine trials in particular, Moodley argues that the risks of participation outweigh individual benefits and, thus, that recruitment will largely rely on contributing to the greater good. However, Moodley warns that the African communitarian nature that supports such selfless acts is quickly giving way to the pervasive influence of Western liberalism arriving from all corners including, ironically, through research processes like informed consent.

The one chapter presented by an African who is not from South Africa comes from noted Cameroonian ethicist Godfrey Tangwa. In chapter 11, he builds on Moodley's discussion of the differences between Western and African approaches with new emphasis on the relationship between health care and business. Whereas they are intimately linked in Western systems, he explains that in Africa, "medicine and commerce are bad companions" (p. 179). Further, it is an African ideal that no one ought to die from need, which is antithetical to the Western approach that the more one needs something, the more they should pay for it in the classic supply and demand relationship. Tangwa purports "to search in the 'junk bag', so to speak, of abandoned or about to be abandoned traditional Africa values and practices to see whether there may not be elements" to inform responses to HIV/AIDS in Africa (p.181). The effort in this chapter goes some distance to meeting this goal, but a more thorough appreciation of Tangwa's wisdom on this topic requires a broader reading of his work. Among Tangwa's greatest gifts is his use of metaphor, such as this quote with which he ends his chapter:

"Ethics is about... being unable to sleep with a quiet conscience should [one's neighbour] happen to die of an ailment whose remedy or part thereof is locked up in one's cupboard (p. 189)."

In chapter 12, the third discussion of HIV vaccine trials takes a different slant, examining the issue of enrolling children in trials. Melissa Stobie, Ann Strode and Cathy Slack present a coherent and timely analysis of the ethical and legal dimensions of the 'best interest standard' for children in South Africa vis-à-vis HIV vaccine trial participation. By drawing on legal frameworks (e.g., the Constitution of the Republic of South Africa) and ethical guidelines (e.g., the South Africa Medical Research Council's Guidelines of Ethics for Medical Research), they illustrate the disjuncture that exists between the paternalistic protection of specific children versus the anticipated generic protection of children as a vulnerable population who deserve special safeguards. This dilemma mirrors the tension between individual and collective good, and between deontological and consequentialist approaches that have been woven throughout the book.

Ultimately, Stobie *et al *recommend that the best interest standard should apply to both individual children and children as a class, and should be used to balance paternalism with empowerment. To further guide practice, they present a range of criteria (e.g., age, needs, child's viewpoint) that should inform this balancing act.

### Revisiting blame: overview of chapter 13

In the final chapter of the anthology, co-editor Loretta M. Kopelman presents yet another approach to the issue of ethics and AIDS in Africa by drawing on historical and biblical ideas embedded in the punishment theory of disease. She critiques this theory which understands people as being culpable for acquiring HIV because of sins (in the religious form) or dubious lifestyles (in the secular form). Ultimately, and not surprisingly, she discredits this approach in both its religious and secular forms and argues that emphasis on this approach to HIV is counterproductive and undermines the compassionate care for those in need.

## Part 2. Reflecting on emerging themes

### The morally dubious response to HIV/AIDS by the South African leadership

A recurring theme throughout the anthology (to the point of redundancy) is critique of the mismanagement of HIV/AIDS by the South Africa political leadership, and President Thabo Mbeki and Minister of Health Manto Tshabalala-Msimang in particular. This repetition can be explained by at least two factors: first, that the issue itself is so dire, and second, that 9 of the 13 chapters are authored by South Africans. However, there are important insights presented to broaden the opinion of readers who may only have heard of Mbeki as a morally-questionable president who queries whether HIV causes AIDS. Rather, we see Mbeki painted as an outstanding leader helping South Africa through the difficult post-Apartheid transition, and for whom AIDS policy has been a bizarre anomaly in an otherwise impressive political record. Various authors provide insight into his baffling stance, drawing most compellingly on explanations that are located within historical contexts of Western domination and imperialism as opposed to explaining away the phenomenon as due to a corrupt or uncaring president. Of course, such explanations do not excuse this behaviour or the profound suffering that it has caused.

To deepen this analysis, we would have appreciated further exploration of the by-products of these (in)actions. For instance, one of us (NN) has found that a central reason for the apathetic response to HIV/AIDS from South Africa youth leaders (in contrast, for instance, to youth leadership during the transition from Apartheid in the last decade) is that statutory youth organizations adopt the policy platforms of their parent organizations. Thus, the ANC's abandonment of HIV/AIDS at the highest level has been mirrored among their youth leaders. However, South Africa is currently experiencing what appears to be a sea-change whereby the morally dubious approaches of President Mbeki and his Minister of Health may be reversing and a new dawn is (possibly) being ushered in for HIV/AIDS policy.

### Insufficient attention to gender concerns

A glaring omission in this book is a sophisticated treatment of the ethical dimensions of gender vis-à-vis AIDS in Africa. This is not to say that gender is not taken up as an issue by several authors, but the analyses are narrow at best (e.g., suggesting that women's disempowerment is primarily due to insufficient education, p. 63). Furthermore, in keeping with earlier comments about the limited engagement with ethics theory, there is an absence of feminist ethics perspectives informing our thinking on these issues.

One final note is that only 3 of the 13 chapters were authored by women. Tallying gender ratios is not always a useful exercise, but in the case of AIDS in Africa where the voices of women have historically been underrepresented, this sort of reflection is essential.

### Recognizing Africa's heterogeneity

The fact that this book has been written largely by African academics is an important contribution for a field that is dominated by commentators from the West. However, it is noteworthy that all of the African authors, with one important exception, are from South Africa and that the focus of many chapters is the South African experience. Indeed, there are enough ethical issues complicating AIDS efforts in South Africa to fill several books, but the stated aim of this anthology is Africa, which leaves room for further reflection.

In a related concern, any discussion of AIDS in Africa risks homogenizing Africa as if it is one culture experiencing one HIV epidemic. This is, of course, fully inaccurate. However, comments such as, "Too little is known about the culture of African sexual practices and the curbs on sexual behaviour that would be conducive to the prevention of AIDS" (p. 63), remind us of the need to explicitly recognize when issues are relevant to Africa broadly (e.g. destructive effects of odious debt) and when not they are not (e.g. links between cultural practices and HIV risk).

### Undervaluing human rights arguments

A peculiar omission is discussion bridging the contribution of human rights with ethics in our thinking about AIDS in Africa. Human rights and ethics share terrain, but can also offer unique and complementary perspectives in analyses of HIV/AIDS. The one time that human rights was invoked in this anthology involved a critique of the "almost inordinate emphasis" on the right to privacy in the management of HIV and how this focus may have inadvertently contributed to further stigmatization of HIV (p. 64). The author then warns about the danger of "rights-talk". This perspective is consistent with other criticisms of human rights-based approaches to HIV of late, especially in the context of testing; that is, whether the voluntary counselling and testing that dominates current thinking and that emerged in the West amidst heightened concern for protection of human rights is an appropriate model for generalized epidemics in Sub-Saharan Africa.

What is important in both of these cases is to avoid throwing the baby out with the bathwater. While there may be shortcomings of overemphasis on the right to privacy, this should not translate into a full-scale discounting of human rights-based approaches to HIV in general. There is a universe of other rights that can contribute to the way that, for instance, opt-out routine testing is implemented, or that can promote a response to HIV that is not individualistic or secretive in nature [6]. In other words, broad statements about the danger of "rights-talk" are not nearly as helpful as more specific critiques of rights that recognize both their usefulness and limits in our response to HIV.

## Part 3. Toward a coherent ethical framework on AIDS in Africa

It is because the co-editors have provided such a useful range of insights on the ethics of AIDS in Africa that we are in the position to reflect on this effort to inform future work. The topics presented in this anthology have been diverse at times, overlapping at others, and have indeed "challenged our thinking" about responses to AIDS in Africa. A useful next step would be the development of a coherent framework that unites these oft-disparate dimensions of the problem; a framework that provides a mechanism for systematically laying out the range of ethical issues facing AIDS in Africa in a way that is accessible yet thorough.

By no means are we trying to be reductionist in our approach to the problem. On the contrary, we are advocating a framework that enables broader reflection on the topics that are receiving attention as well as those that are not. Not only could such an ethics framework provide the basis for identifying a more comprehensive range of issues, but the framework itself would be open to externalist critique for the kinds of perspectives that it excludes, thus expanding our universe of concern even further.

What might such a framework look like? We propose that Benatar's micro/meso/macro framework provides an excellent starting point for mapping out the range of issues from, for instance, micro-level issues with informed consent, as discussed by Moodley and Tangwa, to meso-level concerns regarding the participation of children in HIV vaccine trials, as discussed by Strode *et al*, to macro-level concerns with the impact of globalization on vulnerability to HIV/AIDS, as discussed by Benatar. This architecture serves to reveal not only areas that have been addressed but, importantly, those that have been neglected.

At the micro-level, for instance, we are better able to see that concerns related to the research process have been explored here, but that ethical dimensions of the clinical encounter, especially in this era of treatment scale-up, have been overlooked. Vaccines have been given due attention, but other prevention technologies, such as microbicides and circumcision are equally deserving of ethical scrutiny. Also at the micro-level is an absence of the voice of people living with HIV regarding the ethical dilemmas that they face in their lives. One further omission is an ethical analysis of 'ABC' prevention programming and its usefulness and limits in different settings.

Meso-level concerns with community value systems have been discussed, but topics such as the rationing of scarce ART have not. This is also where we might see ethical analyses of the impact of HIV on health systems and the dilemmas they face in mounting responses, including issues around health care worker migration. The meso-level also serves to shine a light on the dicey concern of cultural practices and their links to HIV.

At the macro-level, global disparities in wealth and health have been highlighted, but rich countries have largely escaped moral scrutiny for their role in both helping and exacerbating the pandemic in Africa through their trade, economic and development policies. The proliferation of HIV/AIDS donors is also a topic for ethical analysis with focus on major players that have emerged in recent years including the Bill and Melinda Gates Foundation and PEPFAR. A final suggestion is to focus on the macro-level issue of global health governance, including the role of UN agencies.

Beyond the gaps illuminated by this particular ethics framework, a welcome contribution to conceptualizing the ethical dimensions of AIDS in Africa would be a critique of this theoretical approach for the kinds of issues it privileges and excludes. One such critique could come from feminist scholars, drawing in particular upon ethics-in-care approaches to thinking about the pandemic and responses to date. The point of these various conceptual framings is not only to give rise to theoretical analysis of issues, but to a programme of empirical research that examines how issues are being played out (and could or should be played out) on the ground.

As noted by Selgelid, the ethics of AIDS in Africa is an area in dire need of study. With *Ethics and AIDS in Africa*, van Niekerk and Kopelman provide us with an excellent springboard for moving toward that goal.

## Competing interests

Both authors are researchers at the Health Economics and HIV/AIDS Research Division (HEARD) at the University of KwaZulu-Natal where Alan Whiteside (lead author on chapters 1 and 2) is the Director. Solomon Benatar (author of chapter 5) was a PhD co-supervisor for Stephanie Nixon.

## Authors' contributions

SN and NN analyzed the book. SN wrote the first draft of the manuscript. Both authors read and approved the final manuscript.
